# Reversible Platelet Aggregation Induced by Low-Temperature Storage in Heparinized Whole Blood Samples

**DOI:** 10.3390/hematolrep17050042

**Published:** 2025-08-22

**Authors:** Yuriko Hayashi, Manato Miyazaki, Ryusuke Kimura, Ririka Arai, Miu Takada, Ayuko Takahashi, Hirokazu Kimura

**Affiliations:** 1 Department of Health Science, Graduate School of Health Sciences, Gunma Paz University, Takasaki-shi 370-0006, Gunma, Japan; hayashi@paz.ac.jp (Y.H.); r-arai@paz.ac.jp (R.A.); t.miumiu501@icloud.com (M.T.); ay-takahashi@paz.ac.jp (A.T.); 2 Faculty of Medical Science and Technology, Gunma Paz University, Takasaki-shi 370-0006, Gunma, Japan; pk3daa@icloud.com; 3 Gunma Prefectural Institute of Public Health and Environmental Sciences, Maebashi-shi 371-0052, Gunma, Japan; kimura-r56@pref.gunma.lg.jp

**Keywords:** heparin, platelet aggregation, pseudothrombocytopenia, fibrinogen, GPIIb/IIIa, docking simulations

## Abstract

**Background/Objectives**: Platelet counts can be affected by storage conditions, potentially leading to pseudothrombocytopenia. The present study aimed to investigate temperature-dependent changes in platelet counts and morphology in whole blood samples anticoagulated with heparin or EDTA. We also examined the molecular mechanism of cold-induced aggregation via integrin GPIIb/IIIa–fibrinogen interaction using established bioinformatics technologies (docking simulation). **Methods**: Peripheral blood was collected from healthy volunteers (*n* = 6) and treated with either heparin or EDTA. The samples were stored at 4 °C, room temperature, or incubated at 37 °C. Platelet counts were measured using an automated hematology analyzer. The morphology of various blood cells in smears was assessed using the May-Grünwald Giemsa staining method. Docking simulations using an available software (HADDOCK 2.4) were performed to evaluate integrin–fibrinogen binding at different temperatures. **Results**: In automated blood cell counting, platelet counts in heparinized blood were significantly decreased under low-temperature conditions (4 °C), but this decrease was restored to levels comparable to those at room temperature upon warming to 37 °C (*p* < 0.05). No significant changes were observed in EDTA-treated samples. Microscopical findings showed platelet aggregation only in heparinized samples at 4 °C, with normal morphology restored upon warming (37 °C). Docking simulations estimated stronger integrin GPIIb/IIIa–fibrinogen binding at 4 °C than at 37 °C (*p* = 0.0286), suggesting temperature-dependent enhancement of molecular interactions. **Conclusions**: These findings indicate that heparin can induce reversible platelet aggregation at low temperatures in whole blood samples, leading to pseudothrombocytopenia. This phenomenon may be mediated by increased integrin GPIIb/IIIa–fibrinogen binding.

## 1. Introduction

Accurate evaluation of platelet counts in whole blood is essential for the diagnosis and management of patients with hemorrhagic diathesis [1,2]. Therefore, it is important to understand how platelets may be affected during the blood collection and specimen handling processes [3,4,5,6]. Some anticoagulants commonly may be used in clinical laboratory testing include ethylenediaminetetraacetic acid (EDTA), sodium citrate, and heparin [7]. Each anticoagulant has specific chemical properties and they may influence test results including platelet counts in whole blood [7]. Of them, EDTA may be widely used for complete blood counts and blood smear examinations [7,8]. However, EDTA-dependent pseudothrombocytopenia (EDTA-PTCP) has been reported, where platelets aggregate or form satellitism, resulting in spuriously low platelet counts despite normal platelet levels in vivo [9,10,11]. In such cases, retesting with alternative anticoagulants such as sodium citrate or heparin is performed, although each has its own limitations [12]. Another anticoagulant, sodium citrate may cause a dilution effect, while heparin can be associated with heparin-induced thrombocytopenia (HIT) or pseudothrombocytopenia caused by cold-induced platelet aggregation [13,14]. Moreover, previous studies have reported that platelet counts in heparinized samples may decrease abnormally when stored at low temperatures during hematological analysis or flow cytometry [15]. This phenomenon is thought to result from increased binding affinity between fibrinogen and platelet integrins, particularly αIIbβ3, under hypothermic conditions, promoting platelet aggregation [16,17,18,19]. Thus, it is hypothesized that the temperature-dependent modulation of integrin-ligand affinity and receptor conformational changes underlie this phenomenon [20,21,22], while the exact mechanism remains unclear.

In light of these circumstances, the present study aimed to examine how temperature affects platelet aggregation in both heparinized and EDTA-anticoagulated blood. Furthermore, by focusing on the interaction between integrin GPIIb/IIIa and fibrinogen, we performed molecular docking simulations to analyze temperature-dependent changes in molecular binding affinity, thereby contributing to a deeper understanding of platelet aggregation mechanisms.

## 2. Materials and Methods

### 2.1. Subjects, Anticoagulation of Whole Blood with Heparin or EDTA

Whole blood was drawn from the peripheral veins of six healthy adult volunteers (mean age 26.5 ± 11.6 years; range, 21–50 years; 1 male and 5 females) after obtaining written informed consent. Whole blood samples were collected via venipuncture into vacuum blood collection tubes pre-filled with anticoagulants using standard phlebotomy techniques [23]. EDTA-treated samples were collected into 5 mL tubes containing dipotassium EDTA (VP-DK050K, Terumo, Tokyo, Japan; final concentration: 1.5 mg/mL). Heparinized samples were collected into 5 mL tubes containing unfractionated sodium heparin (VP-H050K, Terumo, Tokyo, Japan; final concentration: 13 IU/mL). The tubes were gently inverted 5–10 times to ensure homogeneous mixing of the anticoagulant with the blood. Sodium citrate was not included in this study due to its known dilutional effect on blood components, which may interfere with accurate comparison of platelet counts.

### 2.2. Treatment of the Whole Blood and Blood Cell Count Using an Automated Hematology Analyzer Based on the Electrical Impedance Methods

Whole blood samples anticoagulated with either heparin or EDTA were stored at 4 °C for 1 h, followed by incubation at 37 °C for an additional 1 h. In parallel, additional whole blood samples were incubated at room temperature for 1 h and then at 37 °C for another hour. Immediately after incubation, all samples were analyzed using an automated hematology analyzer (XP-300, Sysmex Corporation, Kobe, Japan). This analyzer distinguishes platelets from other blood components, including leukocytes and erythrocytes, based on their size (1–4 µm in diameter) and volume (2–20 femtoliters), according to the specifications provided by the manufacturer (Sysmex Corporation).

### 2.3. Observation of Platelet Morphology in the Peripheral Blood Smears

Blood smears were prepared using the wedge method for both heparinized and EDTA-treated samples immediately after collection, after cold storage, and after warming [24]. Smears were stained with May-Grünwald Giemsa and evaluated for platelet morphology and aggregation [25].

### 2.4. Molecular Docking Simulation

To elucidate the temperature-dependent differences in binding affinity between integrin and fibrinogen, we performed docking simulations using HADDOCK 2.4. The crystal structure of the integrin αIIbβ3–fibrinogen complex (PDB ID: 2VDO) was downloaded from the Protein Data Bank and used in the simulation. Among the 20 resulting clusters, the models from the cluster with the best HADDOCK score were selected as optimal. For these four models, binding affinities (Kd) at 4 °C, 25 °C (room temperature), and 37 °C were calculated using the Prodigy module in HADDOCK.

### 2.5. Statistical Analyses

Statistical analyses were performed using R statistical software (version 4.5.0; R Foundation for Statistical Computing, Vienna, Austria). Platelet counts under three storage conditions were compared using the Friedman test for repeated measures. Exploratory pairwise comparisons were conducted using two-sided Wilcoxon signed-rank tests without multiple testing correction. Data were visualized with boxplots showing the interquartile range (IQR), median (horizontal line), and whiskers (1.5 × IQR). Individual values were overlaid as jittered points. A *p*-value < 0.05 was considered statistically significant.

### 2.6. Ethics Status

This study was conducted with approval from the Ethics Committee of Gunma Paz University (approved number PAZ24-33). Written informed consent was obtained from all participants prior to blood collection.

## 3. Results

### 3.1. Changes in Platelet Counts in Heparinized or EDTA-Anticoagulated Whole Blood Due to Temperature Variations

**Figure 1:**
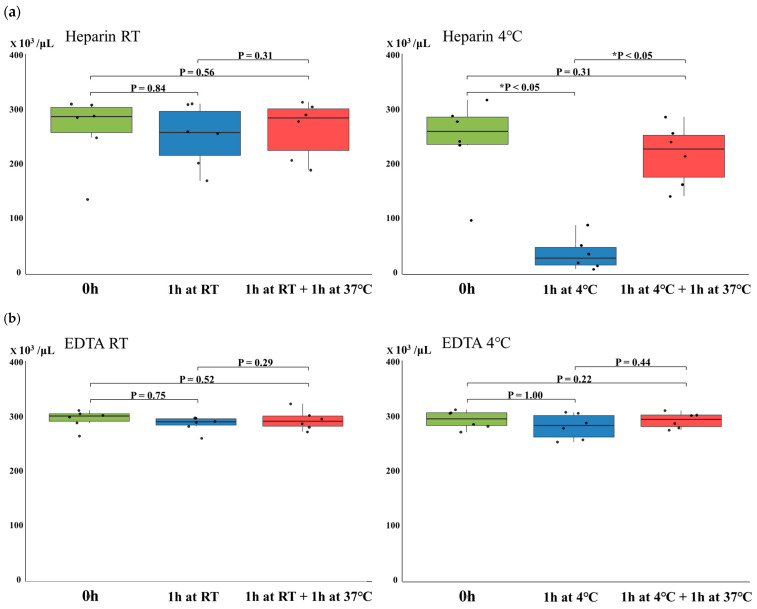
Changes in platelet counts under different storage conditions. (**a**) Heparinized and (**b**) EDTA-anticoagulated blood samples were analyzed under the conditions described in the text. Each boxplot displays the interquartile range (IQR), with the horizontal line indicating the median. The whiskers represent the range within 1.5 × IQR from the lower and upper quartiles. Individual data points (*n* = 6 per group) are shown as jittered dots. Statistical comparison was performed using the Friedman test for repeated measures. Asterisks indicate statistically significant differences (*p* < 0.05).

No statistically significant differences in platelet counts were observed in either heparinized or EDTA-anticoagulated samples when comparing the baseline values to those after storage at room temperature or incubation at 37 °C (Figure 1a,b). In heparinized samples, a marked decrease in platelet count was observed after 1 h of storage at 4 °C, showing a statistically significant reduction compared to the baseline value (*p* < 0.05) (Figure 1a). In contrast, EDTA-anticoagulated samples exhibited no significant changes in platelet count following 1 h of storage at 4 °C (Figure 1b). When the heparinized samples stored at 4 °C for 1 h were subsequently incubated at 37 °C for 1 h, the platelet counts tended to recover to levels close to those observed immediately after collection, with a statistically significant increase (*p* < 0.05) (Figure 1a). On the other hand, EDTA samples showed no significant changes in platelet count either after storage at 4 °C for 1 h or after subsequent incubation at 37 °C (Figure 1b).

### 3.2. Microscopic Findings of the Blood Smears Stained with May-Grünwald Giemsa

**Figure 2:**
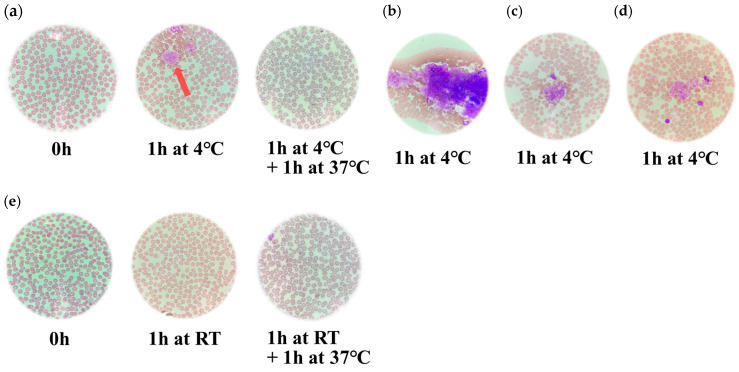
Representative images of platelet aggregation in heparinized whole blood smears under different temperature conditions. (**a**): Heparinized blood smears immediately after collection (0 h), after 1 h at 4 °C, and following incubation at 37 °C. (**b**–**d**): Distinct platelet aggregates observed in different blood smears stored at 4 °C. (**e**): Heparinized blood smears immediately after collection (0 h), after 1 h at room temperature (RT), and following incubation at 37 °C.

To assess platelet morphology, blood smears were prepared from heparinized and EDTA-anticoagulated whole blood samples. Samples were stored at 4 °C for 1 h, followed by incubation at 37 °C for an additional 1 h. In parallel, additional whole blood samples were incubated at room temperature for 1 h. Notable, in heparinized samples, many aggregated platelets were observed after 1 h of storage at 4 °C (Figure 2a–d). Smears of EDTA-anticoagulated whole blood showed no significant morphological changes or platelet aggregation under the same conditions. Platelet aggregates showed irregular morphology, and the number of platelets per aggregate was inconsistent. Moreover, the poorly defined margins of individual platelet membranes rendered it difficult to quantify the number of platelets within each aggregate (Figure 2a–d). Furthermore, in smears prepared after incubation at 37 °C, a marked reduction in platelet aggregation was observed, and individual, non-aggregated platelets were more clearly distinguishable. In contrast, heparinized whole blood samples stored at room temperature exhibited no significant alterations in platelet morphology (Figure 2e).

### 3.3. Evaluation of the Binding Affinities Between Integrin GPIIb/IIIa and Fibrinogen

**Figure 3:**
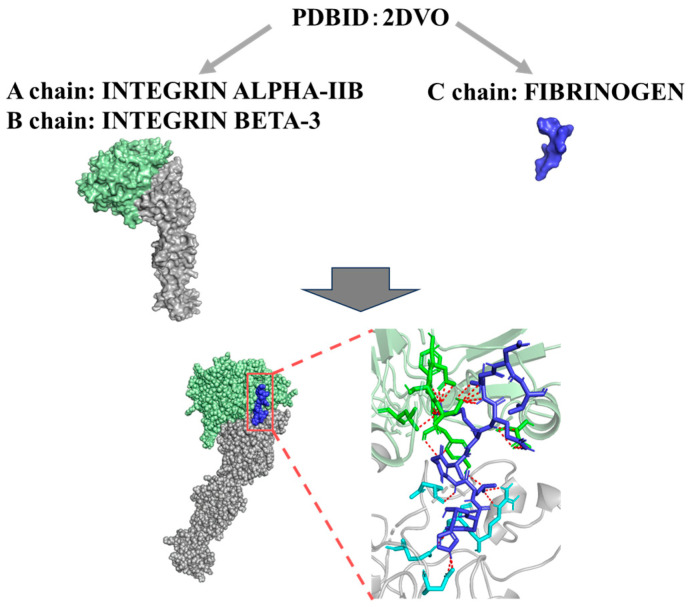
Docking simulations of the Integrin αIIbβ3–Fibrinogen complex using HADDOCK 2.4. The crystal structure of the integrin αIIbβ3–fibrinogen complex (PDB ID: 2VDO) was obtained from the Protein Data Bank and used as input for the simulations.

To evaluate the temperature-dependent changes in the molecular interactions between integrin GPIIb/IIIa and fibrinogen, we evaluated using an advanced docking simulation method. As a result, the binding affinity (Kd) between integrin GPIIb/IIIa and fibrinogen under different temperature conditions was evaluated using molecular docking analyses based on their structural models (Figure 3). At 4 °C, the Kd values for the interaction between integrin GPIIb/IIIa and fibrinogen were consistently lower than those at 37 °C across all models, indicating significantly stronger binding at the lower temperature (*p* = 0.0286) (Table 1). These findings suggest that the interaction between integrin GPIIb/IIIa and fibrinogen is enhanced at lower temperatures, likely due to temperature-dependent conformational changes in the integrin molecule that favor ligand binding.

The table shows the minimum, 25th percentile, median, 75th percentile, and maximum for each temperature group. A statistically significant difference in medians was observed between the groups using the Mann–Whitney U test (*p* = 0.0286).

## 4. Discussion

In the present study, we studied temperature-dependent changes in platelet counts and morphology in whole blood samples anticoagulated with either heparin or EDTA. First, platelet counts in heparinized samples significantly decreased after 1 h of storage at low temperature (4 °C) compared to room temperature (RT) (*p* < 0.05), indicating cold-induced aggregation (Figure 1 and Figure 2). However, when these samples were subsequently incubated at 37 °C for 1 h, platelet counts significantly recovered to near-RT levels (*p* < 0.05), suggesting that the aggregation was reversible. In contrast, EDTA-anticoagulated samples showed no significant changes in platelet counts under any temperature condition, including storage at 4 °C or incubation at 37 °C (Figure 1). Microscopic examination of blood smears revealed marked platelet aggregation in heparinized samples stored at 4 °C, which was largely resolved after warming (Figure 1 and Figure 2). These findings were observed in heparinized samples, whereas no such platelet aggregation was observed in EDTA-anticoagulated blood. Furthermore, to explore the underlying molecular mechanisms, docking simulations were conducted to evaluate the binding affinity between integrin GPIIb/IIIa and fibrinogen. The results demonstrated significantly stronger binding at 4 °C than at 37 °C (*p* = 0.0286), indicating temperature-enhanced ligand binding (Table 1). These findings suggest that cold-induced reversible platelet aggregation in heparinized blood is mediated by increased integrin–fibrinogen interactions, while EDTA provides greater stability under varying temperature conditions.

While sodium citrate is also a commonly used anticoagulant in clinical hematology, it was not included in this study. This was because sodium citrate, being in liquid form, induces a dilutional effect on blood samples, which may alter platelet count values and confound direct comparisons with EDTA and heparin [26]. The primary objective of this study was to examine the temperature-dependent aggregation mechanism specific to heparin, in contrast to the thermally stable profile of EDTA. Nonetheless, incorporating sodium citrate as a third comparator may help generalize the findings in future studies. Heparinized whole blood is known to induce platelet aggregation under hypothermic conditions [27]. Our microscopic analysis further confirmed that this reversible aggregation was only evident in heparinized samples and not in EDTA-treated blood. These findings suggest that the phenomenon is not simply an artifact of sample processing, but a specific interaction modulated by both the anticoagulant type and temperature. This observation may be consistent with a previous report [15].

The anticoagulant effect of heparin is well established and primarily mediated through the enhancement of antithrombin activity [28]. Heparin binds antithrombin via a specific pentasaccharide sequence, inducing conformational changes that increase antithrombin’s affinity for activated coagulation factors, particularly thrombin (factor IIa) and factor Xa [17,29]. By promoting the inactivation of these serine proteases, heparin inhibits thrombin generation and the conversion of fibrinogen to fibrin, ultimately preventing stable clot formation [17]. Additionally, inhibition of thrombin activity suppresses thrombin-mediated platelet activation and aggregation, further contributing to its anticoagulant properties. Interestingly, our results indicate that the cold-induced platelet aggregation observed in only heparinized whole blood occurs independently of this canonical anticoagulant mechanism. Instead, the aggregation appears to be mediated by temperature-sensitive structural changes in platelet membrane proteins, particularly integrin αIIbβ3 (GPIIb/IIIa), which are known to enhance fibrinogen binding when activated.

An alternative mechanism by which heparin induces platelet aggregation involves the formation of complexes between platelet factor 4 (PF4), a positively charged chemokine released from platelet α-granules, and negatively charged heparin molecules [29,30,31]. In certain individuals, these PF4–heparin complexes are recognized as neoantigens, triggering the production of anti-PF4/heparin IgG antibodies [30,32,33]. These antibodies may subsequently bind to FcγRIIa receptors on the platelet surface, leading to platelet activation and excessive aggregation—a pathogenic hallmark of heparin-induced thrombocytopenia (HIT) [30,34]. Notably, this immune-mediated aggregation can occur under physiological temperature conditions [30,32,33]. In contrast, the low-temperature (4 °C)-dependent aggregation observed in the present study is more plausibly attributed to a distinct mechanism involving enhanced molecular interactions between integrin GPIIb/IIIa and fibrinogen, as proposed above (Figure 3). Moreover, in this study, we used unfractionated heparin (UFH), which has a higher molecular weight and broader activity profile compared to low molecular weight heparin (LMWH). While both forms enhance antithrombin activity, UFH is more commonly associated with in vitro platelet aggregation effects, including heparin-induced thrombocytopenia and cold-induced pseudothrombocytopenia [35,36,37]. Future studies may compare UFH and LMWH directly to assess potential differences in temperature-dependent platelet behavior.

Previous studies also suggested that hypothermia decreases membrane fluidity, thereby promoting conformational changes in integrins that facilitate high-affinity binding to fibrinogen [15]. This interaction enables fibrinogen-mediated inter-platelet bridging and aggregation, even in the absence of classical agonists [38]. In addition, cold-induced platelet aggregation may be further influenced by heparin’s physicochemical properties as a highly sulfated, negatively charged polysaccharide [15]. Heparin can interact directly with platelet membranes, modulate local calcium ion dynamics, and potentially enhance the activation state of integrins [39]. Thus, fibrinogen itself may also undergo structural changes at low temperatures that increase its affinity for activated GPIIb/IIIa [40].

Next, to validate this proposed mechanism, we performed molecular docking analyses using structural models of integrin GPIIb/IIIa and fibrinogen under different temperature conditions (4 °C and 37 °C). The underlying mechanism of cold-induced aggregation observed in heparinized blood appears to involve temperature-dependent conformational changes in platelet membrane proteins—particularly integrin αIIbβ3 (GPIIb/IIIa)—which enhance their affinity for fibrinogen. At lower temperatures, reduced membrane fluidity and altered integrin structure likely facilitate spontaneous fibrinogen bridging between platelets. Heparin, as a highly sulfated and negatively charged polysaccharide, may further contribute by modulating the platelet membrane microenvironment or calcium ion availability, thereby promoting integrin activation. These mechanisms are distinct from the canonical antithrombin-mediated anticoagulant effect of heparin and may account for the reversible aggregation observed specifically at 4 °C. The docking simulations revealed significantly higher binding affinity (lower Kd values) at 4 °C than at 37 °C across all models, with a statistically significant difference (*p* = 0.0286) (Table 1). In general, thermodynamic parameters using HADOCK, such as ΔG values below −7 kcal/mol and Kd values below 1 µM, further support the existence of strong and potentially physiologically relevant interactions between integrin GPIIb/IIIa and fibrinogen at lower temperatures [41,42]. Thus, the present results may provide a partly molecular explanation for the cold-induced platelet aggregation observed in heparinized whole blood and highlight the role of temperature-dependent changes in membrane protein conformation in modulating platelet behavior. Moreover, previous reports showed heparin-associated cold-induced platelet aggregation, but these studies were primarily conducted under flow-based in vitro systems simulating physiological conditions [15,38]. In contrast, our study is among the first to quantify this phenomenon under practical clinical specimen storage conditions and to elucidate the molecular mechanisms using docking simulations. This integration of experimental hematological analysis with theoretical molecular modeling represents a novel and comprehensive approach to understanding the effects of temperature on platelet dynamics. In the present study, the interaction between integrin GPIIb/IIIa and fibrinogen was investigated solely in silico using a docking simulation approach. In future studies, in vitro experiments employing antibodies against integrin GPIIb/IIIa and/or fibrinogen may be required to validate these findings.

From a clinical perspective, these findings underscore the importance of strict temperature control during sample handling, particularly for heparinized blood specimens. Failure to recognize temperature-induced, reversible platelet aggregation may lead to misinterpretation of pseudothrombocytopenia and inappropriate clinical decisions [15,34]. Prompt processing and rewarming of samples showing unexpectedly low platelet counts are therefore essential for ensuring accurate hematologic evaluation.

The hematology analyzer used in this study (Sysmex XP-300) identifies platelets based on size and electrical impedance and is incapable of detecting platelet aggregates, which are consequently excluded from the platelet count. Therefore, the observed decrease in platelet counts in heparinized samples at 4 °C is attributable to platelet aggregation rather than true thrombocytopenia. Flow cytometry and platelet aggregometry were not employed in this study, as the objective was to assess the effects of temperature under routine clinical conditions using standard laboratory equipment [43]. Nonetheless, future studies incorporating these advanced techniques would enable more accurate quantification of platelet aggregation and activation at the cellular level.

This study may have several limitations. First, the number of blood samples analyzed was limited, and inter-individual variability—including factors such as age, sex, and baseline platelet function—could not be fully assessed. Second, although docking simulations provide mechanistic insights, they rely on static theoretical models that do not replicate the full complexity of in vivo conditions. Future studies should validate these findings using complementary approaches, including real-time platelet function assays and in vivo models. Additionally, further investigations should examine the effects of heparin concentration, storage duration, and calcium ion (Ca^2+^) levels on platelet aggregation behavior under hypothermic conditions. Next, although our study focused on the integrin GPIIb/IIIa–fibrinogen interaction as a primary mechanism of cold-induced platelet aggregation, other platelet activation pathways—such as collagen–GPVI, thrombin–PAR, or von Willebrand factor (vWF)–GPIb interactions—may also contribute under hypothermic conditions. These alternative mechanisms were not explored in the present study and should be investigated in future research to obtain a more comprehensive understanding of temperature-dependent platelet aggregation.

As the majority of participants in this study were female (5 out of 6), potential sex-related differences in platelet reactivity or integrin expression could not be evaluated. Although sex differences in platelet function have been reported in previous studies, our sample size was insufficient to assess such effects. Future investigations with a larger and more gender-balanced cohort are warranted to explore the impact of sex on temperature-dependent platelet aggregation and related molecular interactions [44].

Moreover, to further validate our findings in a physiological context, we propose using murine models in which cold-induced thrombocytopenia or platelet aggregation can be induced and assessed. For example, transgenic mouse models with fluorescently labeled platelets (e.g., PF4-Cre; Rosa26-tdTomato) could be utilized to visualize platelet aggregation in vivo under hypothermic conditions using intravital microscopy. Alternatively, wild-type mice exposed to controlled cold environments, followed by platelet count monitoring and histological examination of organs (e.g., lungs, spleen), may provide insight into systemic platelet behavior under low temperatures. Such models would allow direct observation of temperature-dependent platelet dynamics and help evaluate the role of fibrinogen and integrin GPIIb/IIIa in vivo.

In conclusion, our results suggest that the temperature-dependent decrease in platelet count observed in heparinized whole blood is attributable to reversible platelet aggregation mediated by enhanced integrin GPIIb/IIIa–fibrinogen interactions at low temperatures. This aggregation is distinct from heparin’s classical anticoagulant effect and reflects structural and thermodynamic changes at the molecular level. The findings not only provide mechanistic insight into cold-induced platelet aggregation but also emphasize the clinical importance of temperature control in blood sample handling and analysis.

## Figures and Tables

**Table 1:** Temperature-dependent binding affinities between Integrin αIIbβ3 and Fibrinogen.

	Model	ΔG (kcal mol^−1^)		Kd (M) at °C	
4 °C	Model 1	−8.9		8.9 × 10^−8^	
	Model 2	−9.4		4.0 × 10^−8^	
	Model 3	−9.6		2.8 × 10^−8^	
	Model 4	−9.2		5.5 × 10^−8^	
37 °C	Model 1	−8.9		5.0 × 10^−7^	
	Model 2	−9.4		2.4 × 10^−7^	
	Model 3	−9.6		1.8 × 10^−7^	
	Model 4	−9.2		3.2 × 10^−7^	
	Minimum	0.25	Median	0.75	Maximum
4 °C	2.8 × 10^−8^	3.7 × 10^−8^	4.8 × 10^−8^	6.4 × 10^−8^	8.9 × 10^−8^
37 °C	1.8 × 10^−7^	2.3 × 10^−7^	2.8 × 10^−7^	3.7 × 10^−7^	5.0 × 10^−7^
				*p*-value = 0.0286	

Abbreviations: ΔG, Gibbs free energy change; Kd, equilibrium dissociation constant.

## Data Availability

The raw data supporting the conclusions of this article will be made available by the authors on request.
